# Catheter ablation of atrial flutter in an adult with a univentricular heart, common atrium, and single atrioventricular valve: a case report—‘complex things don’t always require a complex solution’

**DOI:** 10.1093/ehjcr/ytae666

**Published:** 2024-12-16

**Authors:** Martín Ortíz-Avalos, Silvia Melissa Galindo-Garza, Chris Keith Chavez-Collazos, Elias Noel Andrade-Cuellar, Gerardo Rodríguez-Diez

**Affiliations:** Cardiac Electrophysiology, National Medical Center ‘November 20th’, ISSSTE, Av. Felix Cuevas #540, Col. Del Valle Del. Benito Juarez, C.P. 03100 Mexico City, Mexico; Cardiac Electrophysiology, National Medical Center ‘November 20th’, ISSSTE, Av. Felix Cuevas #540, Col. Del Valle Del. Benito Juarez, C.P. 03100 Mexico City, Mexico; Cardiac Electrophysiology, National Medical Center ‘November 20th’, ISSSTE, Av. Felix Cuevas #540, Col. Del Valle Del. Benito Juarez, C.P. 03100 Mexico City, Mexico; Cardiac Electrophysiology, National Medical Center ‘November 20th’, ISSSTE, Av. Felix Cuevas #540, Col. Del Valle Del. Benito Juarez, C.P. 03100 Mexico City, Mexico; Universidad Nacional Autónoma de México, Escolar 411A, Copilco Universidad, Coyoacán, C.P. 04360 Mexico City, Mexico; Cardiac Electrophysiology, National Medical Center ‘November 20th’, ISSSTE, Av. Felix Cuevas #540, Col. Del Valle Del. Benito Juarez, C.P. 03100 Mexico City, Mexico

**Keywords:** Atrial flutter, Univentricular heart, Common atrium, Catheter ablation, Cavo-annular isthmus, Congenital heart disease, Case report

## Abstract

**Background:**

The ‘univentricular’ heart encompasses a variety of congenital cardiac defects characterized by a single functional ventricle and an underdeveloped ventricular chamber. Surgical intervention, typically in infancy or childhood, aims to regulate pulmonary blood flow volume. In adulthood, untreated patients may experience limitations in physical activity and elevated morbidity due to persistent cyanosis and arrhythmias, notably after the Fontan procedure.

**Case summary:**

A 38-year-old Mexican man with an unrepaired morphologically right single ventricle and a common atrium presented with palpitations. Diagnostic imaging revealed a hypertrophic systemic single ventricle with severe atrioventricular valve regurgitation and pulmonary stenosis. Despite ongoing anticoagulant and beta-blocker therapy, persistent symptoms prompted catheter ablation guided by CARTO-Merge^®^, a function that overlays computed tomography or magnetic resonance imaging onto a CARTO^®^ electroanatomical map. Ablation along the cavo-annular isthmus was successfully performed, achieving arrhythmia termination. Post-ablation, the patient developed sinus rhythm and second-degree atrioventricular block, necessitating the implantation of an epicardial pacemaker.

**Discussion:**

Atrial flutter ablation in univentricular hearts without prior surgery is rare. Such patients are predisposed to post-Fontan arrhythmias, often requiring intervention due to increased morbidity. Atrial flutter arises from scarred post-surgery regions, necessitating careful assessment and management. Our case demonstrates successful ablation in a complex congenital heart condition, highlighting the importance of comprehensive imaging, understanding arrhythmia mechanisms, and meticulous procedural techniques for optimal outcomes.

**Learning points**

- Ablation of atrial flutter in patients with univentricular hearts without prior surgical intervention is rare, suggesting the need for careful consideration and specialized approaches in managing arrhythmias in this population.
- In complex congenital heart diseases, ablation of arrhythmias can be challenging. A deep understanding of the morphological features of the heart condition as well as the mechanism of the arrhythmia are key to successful ablation.

## Introduction

The term ‘univentricular’ heart encompasses a broad spectrum of congenital cardiac defects characterized by a single functional ventricle and a rudimentary or hypoplastic second ventricular chamber.^1^ Most patients undergo surgery during infancy or childhood, primarily to regulate pulmonary blood flow. Those who reach adulthood without corrective surgery may retain systolic function of the single ventricle, but due to persistent cyanosis, they often experience physical activity limitations, elevated ventricular filling pressures, and increased brain natriuretic peptide (BNP) levels.^2^

Arrhythmias are a frequent and significant issue in patients with a single ventricle, particularly after Fontan palliation, and contribute to increased morbidity and mortality within this population.^3^ Catheter ablation of atrial flutter in Fontan circulation patients poses unique challenges due to the complex and heterogeneous substrates for arrhythmia, compounded by vascular access issues in patients with total cavopulmonary connections.^4^

The case presented here involves an adult with an unrepaired single ventricle, a common atrioventricular valve, and a common atrium, who successfully underwent catheter ablation for typical atrial flutter.

## Summary figure

**Figure:**
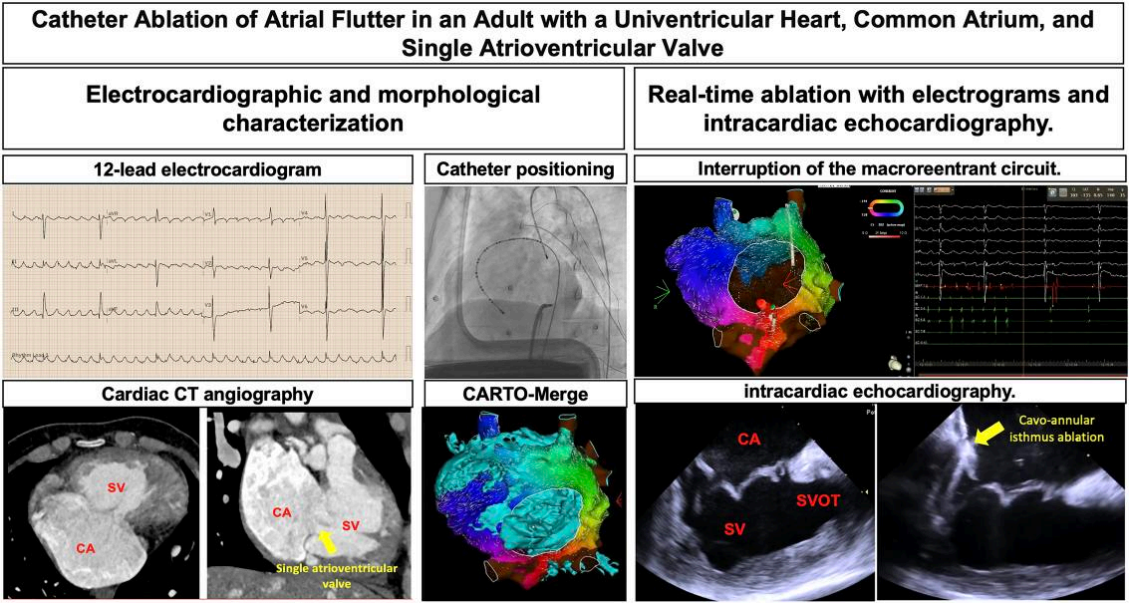


## Clinical case

**Figure 1:**
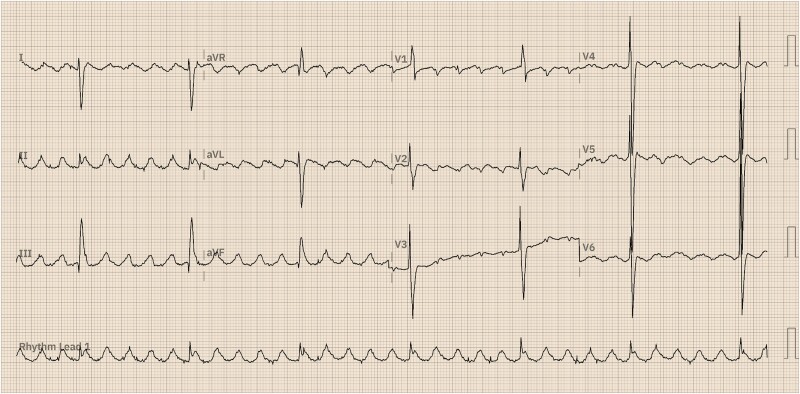
A 12-lead electrocardiogram was recorded at 10 mm/mV and 25 mm/s, revealing atrial flutter with a ventricular rate of 42 beats/min, a flutter cycle length of 290 ms, and right ventricular hypertrophy.

**Figure 2:**
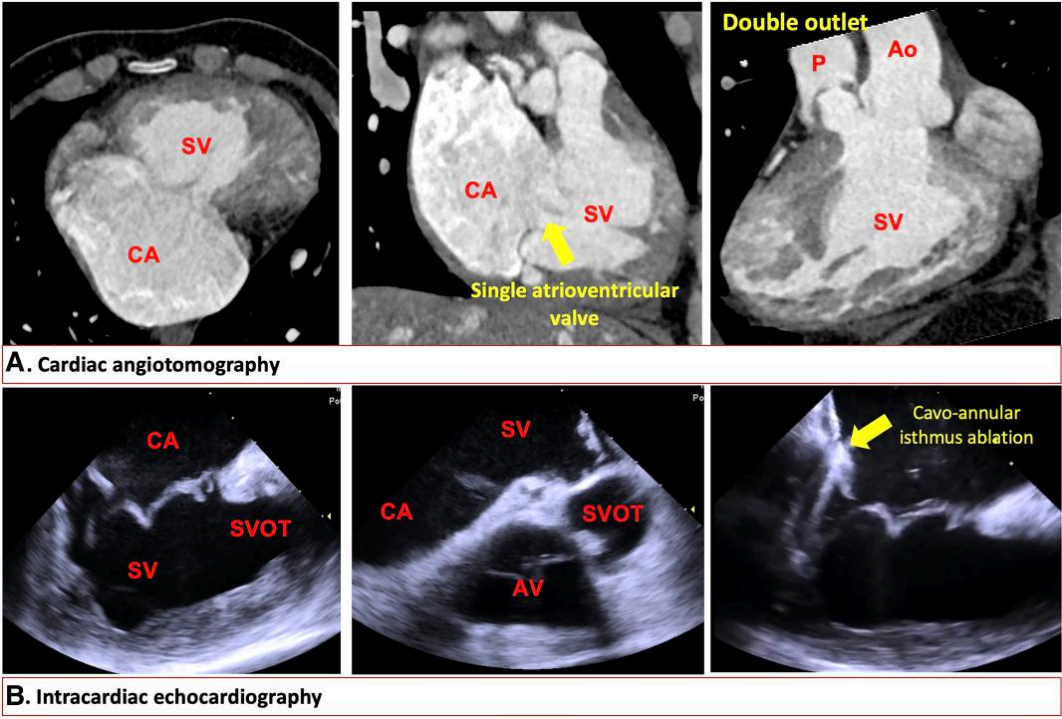
Cardiac computed tomography angiography and intracardiac echocardiography. (*A*) Cardiac computed tomography angiography reveals a morphologically right systemic single ventricle and a dilated common atrium, along with the absence of an interatrial septum and a single atrioventricular valve. The single ventricle exhibits a double-outlet configuration. (*B*) Intracardiac echocardiography. In the home view, the common atrium, single ventricle, and single atrioventricular valve are visualized. The moment of cavo-annular isthmus ablation guided by intracardiac echocardiography is also depicted. CA, common atrium; SV, single ventricle; P, pulmonary artery; Ao, ascending aorta; SVOT, single ventricle outflow tract; AV, aortic valve.

A 38-year-old male with a morphologically right single ventricle was referred to the cardiac electrophysiology clinic for the evaluation of palpitations. The patient had not undergone any prior surgical repair, as severe pulmonary stenosis during childhood had protected him from excessive pulmonary blood flow. During the physical examination, oxygen saturation was measured at 60% in ambient air, with a report of nocturnal oxygen use at home. Blood pressure was 100/60 mmHg, and heart rate was 42 b.p.m. Cardiac auscultation revealed a split and intensified S2, along with a holosystolic murmur over the pulmonary area and clubbing of the fingers. The 12-lead electrocardiogram (*Figure 1*) showed an atrial flutter rhythm, characterized by negative F-waves in V1 and positive waves in leads II, III, and aVF, with a flutter cycle length of 290 ms. Transthoracic echocardiography and cardiac computed tomography (CT) angiography demonstrated situs solitus and a common atrium due to the absence of an interatrial septum, with dimensions of 75 × 97 mm. Regarding venous return, the right superior vena cava drained into the right portion of the common atrium, while the left superior vena cava, inferior vena cava, and pulmonary veins connected to the roof of the left portion of the common atrium. The single ventricle had an inlet via a common atrioventricular valve, which exhibited severe regurgitation, with an annulus measuring 67 mm. The morphologically right single ventricle was hypertrophied, with a diastolic diameter of 100 mm and a systolic diameter of 74 mm, along with the anterior and posterior wall thicknesses of 5 mm each. Additionally, there was significant apical trabeculation, with a preserved ejection fraction (55%) and a global longitudinal strain of −21%. Regarding ventriculo-arterial connections, the single ventricle had a double-outlet configuration, with the aorta positioned to the right and the pulmonary artery to the left. The aortic valve was tricuspid, with no evidence of stenosis or regurgitation. In contrast, the pulmonary valve was bicuspid and exhibited severe stenosis. Finally, the coronary arteries had independent origins, with the right coronary artery and the left coronary trunk arising from the left sinus of Valsalva (*Figure 2A* and Supplementary material online, *Videos S1* and *S2*).

The patient did not present with any muscular, skeletal, ophthalmological, or vascular anomalies suggestive of a congenital syndrome. The patient reported experiencing palpitations and paroxysmal episodes of dyspnoea for 1 year and had been on anticoagulant therapy. The patient was classified as New York Heart Association (NYHA) functional Class II. Due to persistent symptoms despite ongoing treatment, an ablation procedure with electroanatomical mapping using CARTO-Merge^®^ (Biosense Webster) was scheduled.

**Figure 3:**
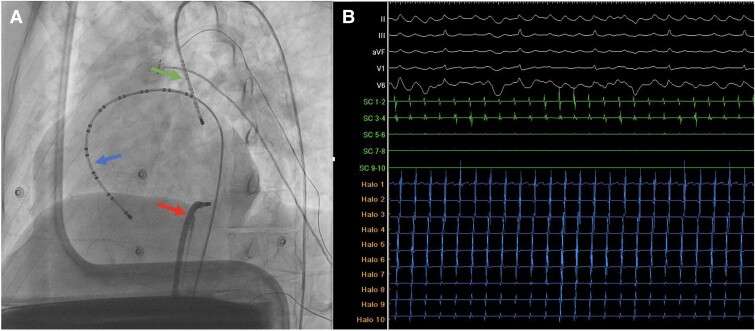
(*A*) Catheter positioning: duodecapolar catheter (Halo, blue arrow), decapolar catheter at the entrance of the superior vena cava (green arrow), and ablation catheter at the cavo-annular isthmus (red arrow) in the left anterior oblique fluoroscopy view. (*B*) Endocardial electrograms from the duodecapolar Halo catheter, decapolar catheter at the upper wall of the single atrium (coronary sinus [CS] 1–4), and surface electrocardiogram. The activation sequence of the single atrium is noted, beginning at CS 1–2 and ending at Halo 1 (counterclockwise activation).

The procedure was performed under conscious sedation. A puncture was made in the left subclavian vein, and a decapolar catheter was advanced through the left superior vena cava to the upper wall of the common atrium. Through the right femoral vein, a steerable duodecapolar catheter (Steerable Duo-decapolar Mapping Catheter) was advanced and positioned on the lateral wall and cavo-annular isthmus (*Figure 3A*). The activation sequence of the atrial flutter was recorded, confirming a counterclockwise activation pattern (*Figure 3B*). After several attempts, cannulation of the coronary sinus was unsuccessful. Following baseline measurements, with a flutter cycle length of 304 ms, entrainment manoeuvres were performed during the atrial flutter. Continuous atrial stimulation at 280 ms from the cavo-annular isthmus resulted in flutter acceleration to the pacing frequency, with no changes observed in the surface electrocardiogram F-wave morphology. The post-stimulation interval was 316 ms, confirming involvement of the cavo-annular isthmus. Post-stimulation intervals along the isthmus matched the flutter cycle length, and concealed entrainment was observed (see Supplementary material online, *Figure S1*).

**Figure 4:**
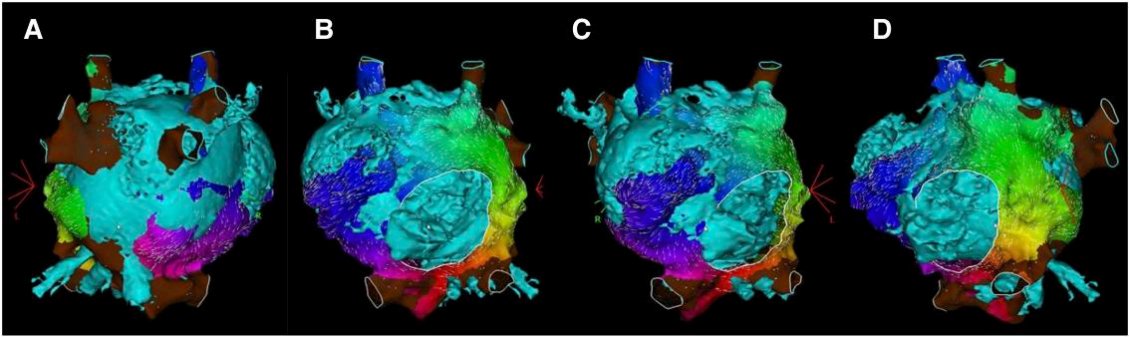
CARTO-Merge^®^ (biosense webster): (*A*) posteroanterior view, (*B*) anteroposterior view, (*C*) right anterior oblique view, and (*D*) left anterior oblique view. The aneurysmal common atrium with a single atrioventricular valve is shown. The right and left superior vena cava drain into the roof of the right and left portions of the common atrium, respectively. The inferior vena cava drains into the left portion of the common atrium. All four pulmonary veins drain into the left portion of the posterior wall of the common atrium.

**Figure 5:**
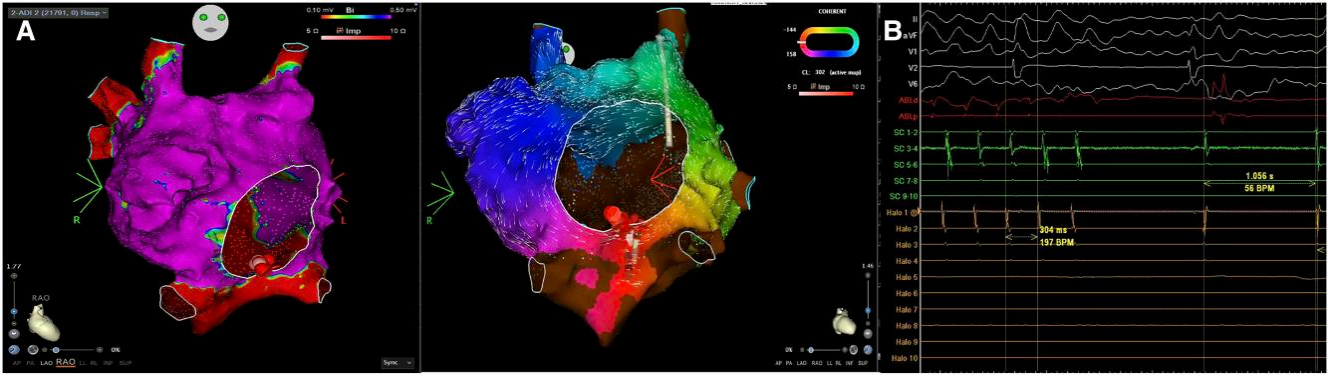
Voltage and activation map of the common atrium. (*A*) The ablation catheter is shown in the isthmus, with red spheres indicating the ablation points. (*B*) Real-time electrograms during ablation showing the moment when the macroreentrant circuit is interrupted, transitioning into sinus bradycardia rhythm.

Through the left femoral vein, an intracardiac ultrasound probe was advanced to the mid-atrium to obtain an image resembling the home view (*Figure 2B* and Supplementary material online, *Video S3*). Through the right femoral vein, the OCTARAY™ mapping catheter (Biosense Webster) was introduced using the bidirectional CARTO VIZIGO™ guiding sheath (Biosense Webster) to perform electroanatomical mapping of the common atrium. CARTO-Merge^®^ was employed to overlay CT images onto the electroanatomical map, enabling precise visualization of the aneurysmal common atrium and facilitating accurate identification of the cavo-annular isthmus and the common atrioventricular valve. The activation map of the common atrium (*Figure 4*, CARTO 3 System) demonstrated a counterclockwise rotation around the common atrioventricular valve (see Supplementary material online, *Video S4*). With the diagnosis of atrial flutter involving the common atrioventricular valve, an ablation line was created along the cavo-annular isthmus (red spheres in *Figure 5A*), successfully terminating the arrhythmia (*Figure 5B* and Supplementary material online, *Video S5*).

The total duration of the ablation procedure was 1 h and 25 min. Complete success was determined by the termination of atrial flutter and the creation of an ablation line along the cavo-annular isthmus (*Figure 5* and Supplementary material online, *Video S5*). A conduction time through the isthmus was determined, measured from the ablation catheter to the Halo catheter, being 196 ms, with a linear activation pattern from proximal to distal (see Supplementary material online, *Figure S2*), suggesting complete conduction block across the isthmus.

The patient exhibited variable atrioventricular conduction from the outset, with a flutter cycle length of 304 ms. After the ablation, the patient presented with sinus rhythm at 65 beats/min (see Supplementary material online, *Figure S3*) and intermittent second-degree Mobitz I atrioventricular block. Therefore, it was decided to implant a bicameral epicardial pacemaker, with the atrial lead positioned at the junction of the superior vena cava and the right atrium, achieving a capture threshold of 0.5 V at 0.6 ms, sensing >5 mV, and a lead impedance of 450 Ω. The ventricular lead was positioned on the inferior surface of the single ventricle, achieving a capture threshold of 0.5 V at 0.6 ms, a sensing amplitude of 2.7 mV, and an impedance of 340 Ω. The device was programmed in the dual-chamber pacemaker mode (DDD) (see Supplementary material online, *Figure S4*). The patient was discharged without antiarrhythmic medication or anticoagulation. At the 10-month follow-up, the patient remained free from flutter recurrence, with ventricular pacing above 99% according to the device evaluation.

## Discussion

To our knowledge, this represents the first reported case in the literature of catheter ablation of typical atrial flutter in a univentricular heart with a single atrium and a common atrioventricular valve, without prior surgical intervention. Most of these arrhythmias typically arise after the Fontan procedure, where prolonged haemodynamic stress often leads to significant abnormalities in the atrial myocardium, predisposing it to the formation of various reentrant circuits around scarred regions within the atria.^5^

Cardiac rhythm irregularities present a common challenge in patients with univentricular hearts, particularly after palliative surgical procedures, with intra-atrial reentrant tachycardia and atrial fibrillation being the most frequently encountered arrhythmias in this patient population.^6,7^ Patients who develop these arrhythmias are at heightened risk for complications, including atrial thrombosis, atrial dilation, heart failure, ventricular dysfunction, hospitalizations, and sinus node dysfunction.^7,8^

Our patient presented with a complex congenital heart disease, a morphologically right single ventricle, associated with a common atrium dilated due to the absence of an interatrial septum. Severe pulmonary stenosis, present since childhood, protected the patient from excessive pulmonary blood flow but led to pressure overload, resulting in hypertrophy of the single ventricle with preserved systolic function. Severe regurgitation of the common atrioventricular valve contributed to atrial dilation and structural remodelling. We believe that the atrial flutter developed by our patient is a consequence of atrial remodelling secondary to chronic dilation and altered atrial filling pressures. This type of arrhythmia further exacerbates haemodynamic dysfunction, reducing effective cardiac output and predisposing the patient to atrial thrombus formation.^7^

It has been described that in patients with septal defects, the His bundle may be displaced towards the posterior wall or the coronary sinus, or it may even be absent.^4^ The patient presented with variable atrioventricular conduction from the outset, indicating different degrees of nodal block due to atrial flutter. During the mapping process, efforts were made to locate the His bundle and avoid its ablation; however, the His bundle could not be identified, even after the procedure. Another difficulty we encountered was in cannulating the coronary sinus. Although CT confirmed the presence of a coronary sinus, it was not possible to cannulate it with a standard decapolar catheter due to the complex anatomy and venous malformations in our patient. These structural anomalies can complicate the catheter’s advancement along the usual anatomical pathway, preventing effective cannulation. CARTO-Merge^®^ was used in this case to overlay CT imaging onto the electroanatomical map, enabling precise alignment of anatomical and electrical data. This facilitated a more detailed visualization of the patient’s unique anatomy, including the aneurysmal common atrium and its vascular connections, ensuring accurate localization of the cavo-annular isthmus as the target site for ablation.

Our patient had an atrial flutter cycle length of 304 ms, which increased the likelihood of requiring permanent pacemaker implantation after ablation, as suggested by a clinical trial that evaluated the cycle length of cavotricuspid isthmus-dependent atrial flutter. This study identified a cycle length >273 ms as the cut-off value for predicting the need for permanent pacemaker implantation;^9^ however, no established values exist for patients with a single atrium. Our patient developed intermittent second-degree Mobitz I atrioventricular block following the ablation, prompting the decision to implant an epicardial pacemaker. The patient had no history of prior thrombo-embolic events. During the procedure, atrial flutter was successfully terminated, and a pacemaker was implanted via the epicardial approach. As a result, the patient was discharged without the need for anticoagulation therapy. Ten months after the procedure, the patient remains free of atrial flutter recurrence.

The procedure was performed at a medical centre with a cardiac electrophysiology service boasting 25 years of experience, where an average of 250 ablations with electroanatomical mapping are conducted annually. This case underscores the complexity and challenges faced by patients with complex congenital heart disease, particularly those with a univentricular heart, in managing arrhythmias such as atrial flutter. Catheter ablation targeting the cavo-annular isthmus can be safely and effectively performed, achieving termination of atrial flutter without major complications. This case report highlights the importance of an individualized approach, based on a thorough morphological and functional understanding, which can improve clinical outcomes in this patient population. The long-term success observed at the 10-month follow-up reinforces the feasibility of ablation as a therapeutic option for patients with similar characteristics.

## Lead author biography


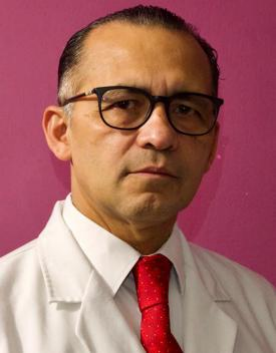


Martín Ortíz-Avalos is a Medical Graduate from the Juárez Autonomous University of Tabasco. He later specialized in Cardiology and Cardiac Electrophysiology at the National Autonomous University of Mexico at the 20 de Noviembre National Medical Center. He served as the President of SOMEEC (Mexican Society of Electrophysiology and Cardiac Stimulation) and currently holds the position of Adjunct Professor in the advanced course on Cardiac Electrophysiology at the 20 de Noviembre National Medical Center. His primary areas of interest include complex cardiac arrhythmias, physiological pacing, and ventricular arrhythmias.

## Supplementary Material

ytae666_Supplementary_Data

## Data Availability

The data underlying this article are available in the article and in its online Supplementary material.

## Notes

**Consent:** Informed consent was obtained from the patient in accordance with COPE guidelines.

**Funding:** No funding was received to assist with the preparation of this manuscript. The authors have reported that they have no financial interests to disclose.
