# Supplementary material for: Catheter ablation of atrial flutter in an adult with a univentricular heart, common atrium, and single atrioventricular valve: a case report—‘complex things don’t always require a complex solution’
Source: Eur Heart J Case Rep. 2024 Dec 16;9(1):ytae666. doi: 10.1093/ehjcr/ytae666 (PMC11733771; doi:10.1093/ehjcr/ytae666)
Supplement: ytae666_Supplementary_Data [file ytae666_supplementary_data.zip › Supplementary Captions.docx]

**Supplementary Material:**
**Figure S1:** Concealed entrainment. Return cycle of 316 ms. Catheter positions: duodecapolar catheter (Halo, blue arrow), decapolar catheter at the entrance of the superior vena cava (green arrow), and ablation catheter at the cavo-annular isthmus (red arrow).

**Figure S2:** Trans-isthmic conduction. Ablation catheter to Halo catheter of 196 ms, with a linear activation pattern from proximal to distal (green arrow). Catheter positions: duodecapolar catheter (Halo, blue arrow), decapolar catheter at the entrance of the superior vena cava (green arrow), and ablation catheter at the cavo-annular isthmus (red arrow)

**Figure S3:** A 12-lead electrocardiogram post-ablation.
**Figure S4:** A 12-lead electrocardiogram (A) and posteroanterior chest radiograph (B) after placement of a biventricular epicardial pacemaker.

**Video S1:** Cardiac CT angiography. 2D reconstruction, posterior-anterior view.
**Video S2:** Cardiac CT angiography. 2D reconstruction, right oblique view.
**Video S3:** Intracardiac echocardiography.
**Video S4:** Activation map of the common atrium showing counterclockwise rotation around the common atrioventricular valve.
**Video S5:** Activation map with vector and velocity information integration, showing counterclockwise rotation around the common AV valve. The moment when the macroreentrant circuit is interrupted and transitions to sinus bradycardia is observed.
