# Supplementary figures and images for: Catheter ablation of atrial flutter in an adult with a univentricular heart, common atrium, and single atrioventricular valve: a case report—‘complex things don’t always require a complex solution’
Source: Eur Heart J Case Rep. 2024 Dec 16;9(1):ytae666. doi: 10.1093/ehjcr/ytae666 (PMC11733771; doi:10.1093/ehjcr/ytae666)

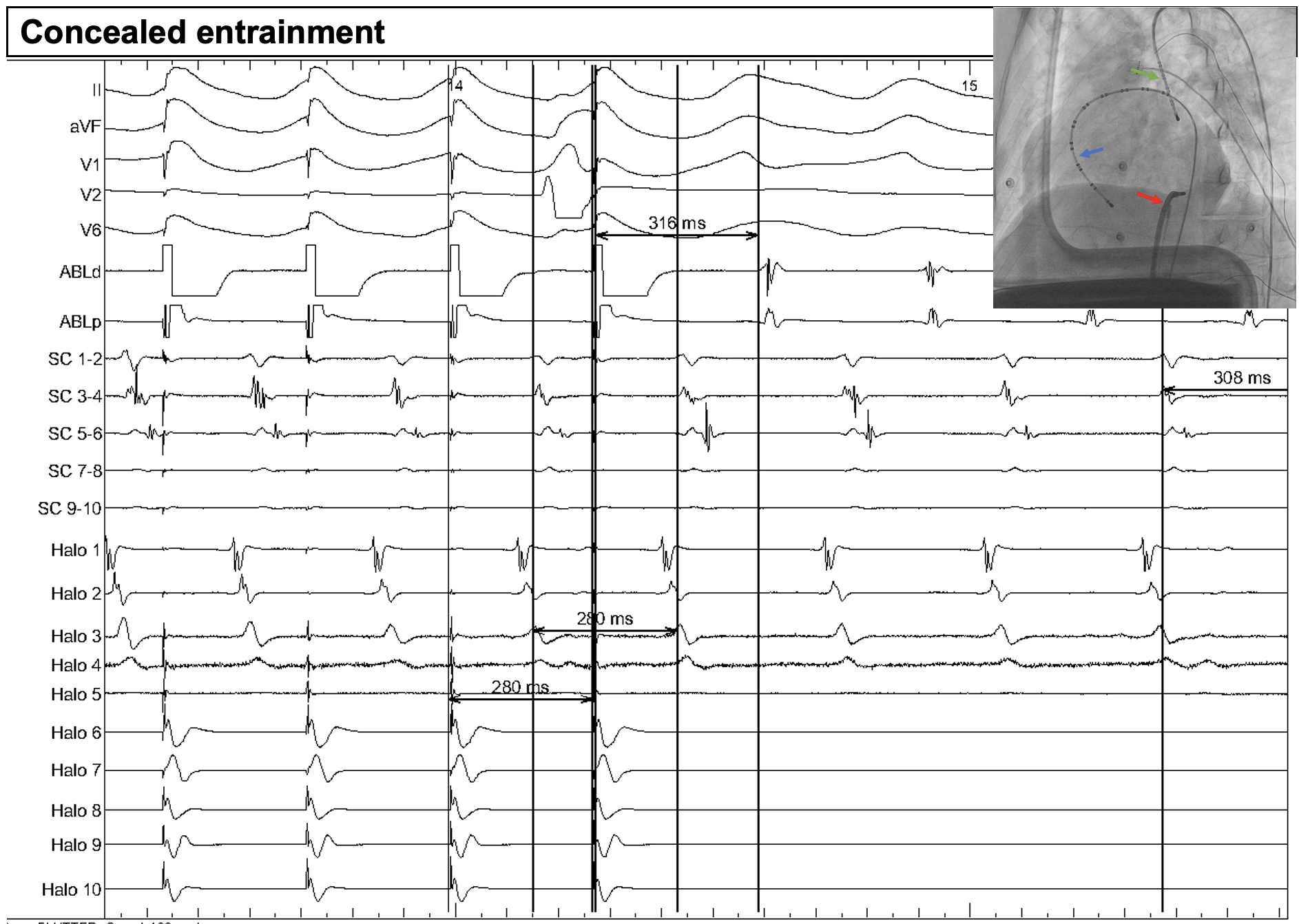

Supplement: ytae666_Supplementary_Data [file ytae666_supplementary_data.zip › Figure S1.jpg]

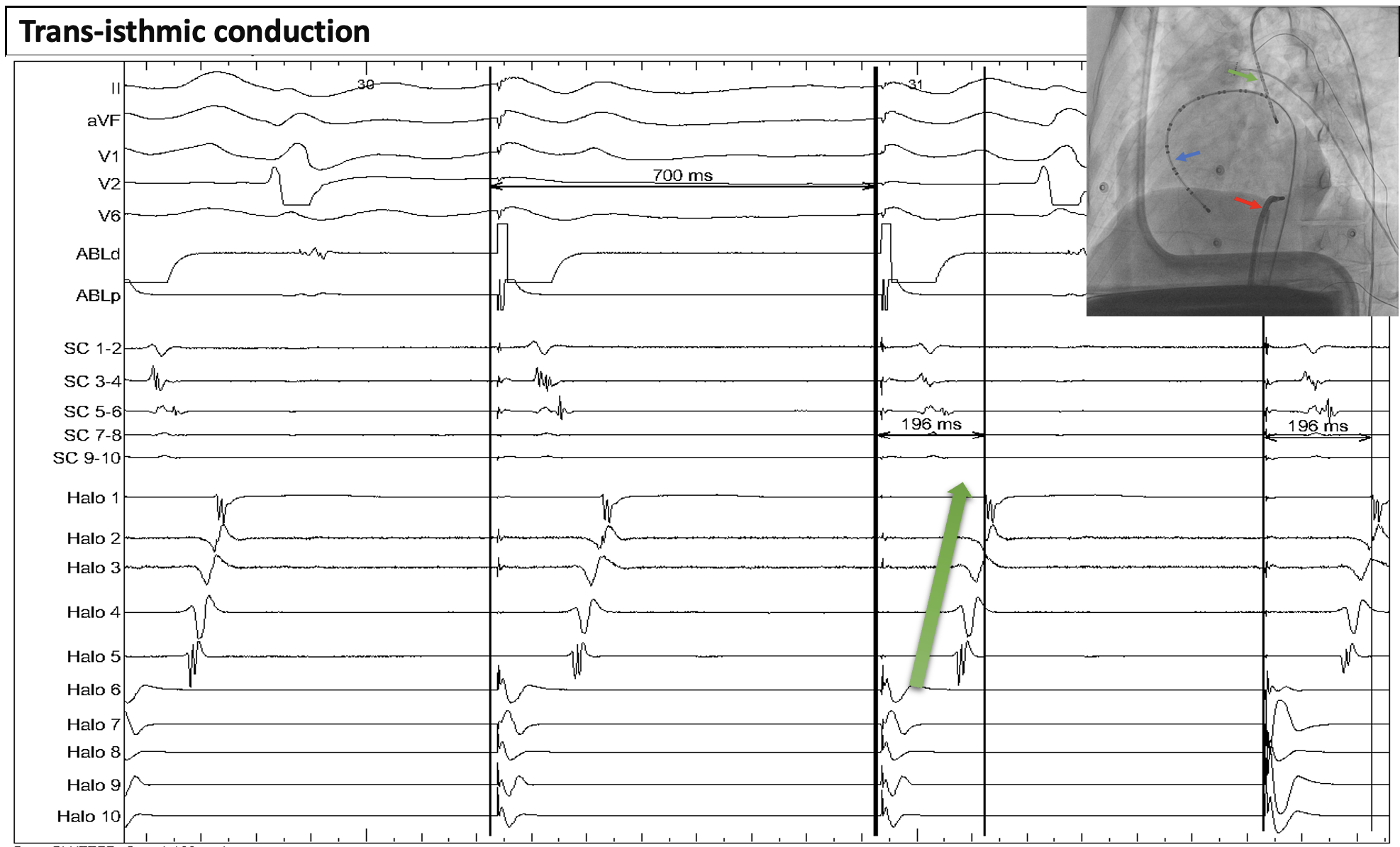

Supplement: ytae666_Supplementary_Data [file ytae666_supplementary_data.zip › Figure S2.jpg]

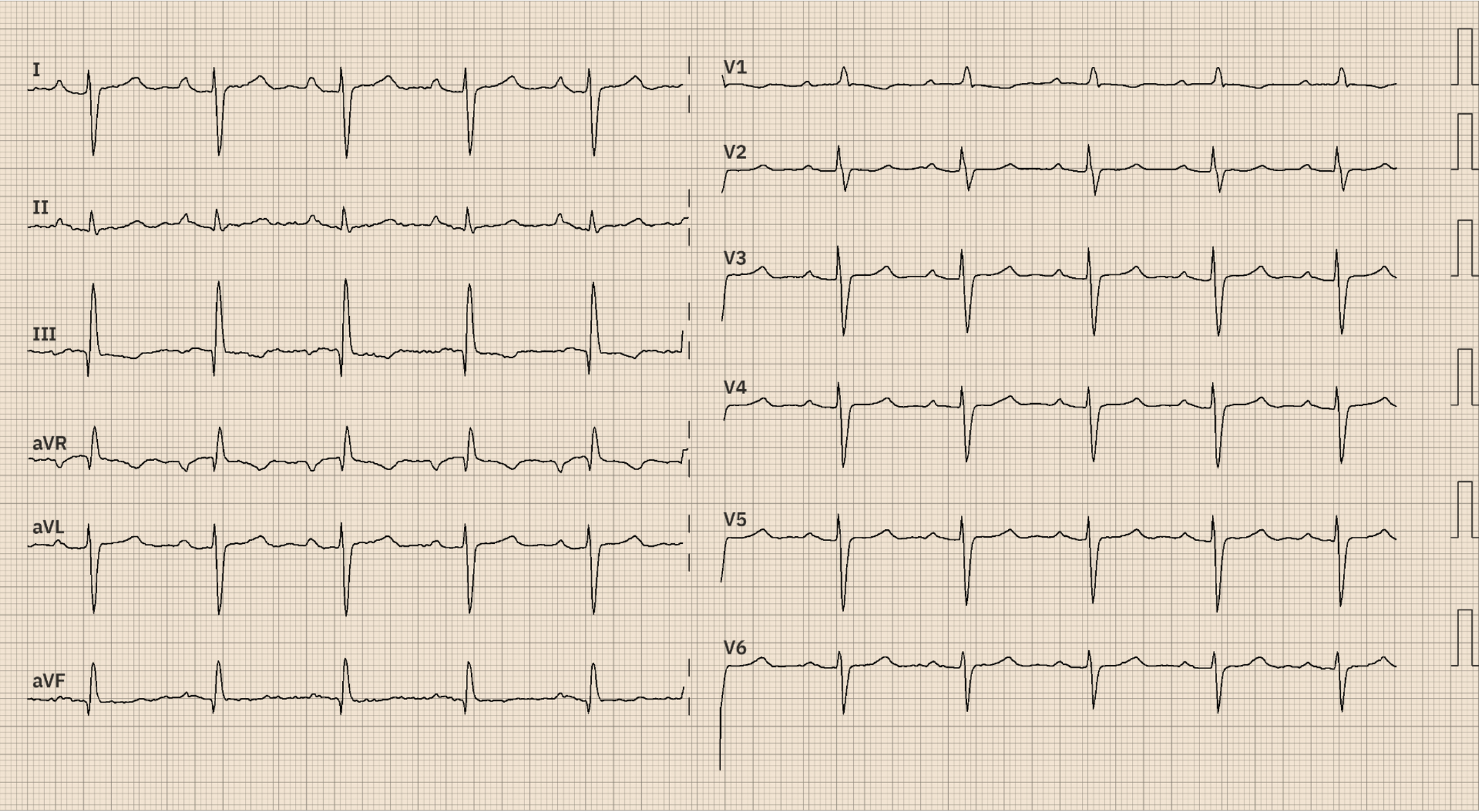

Supplement: ytae666_Supplementary_Data [file ytae666_supplementary_data.zip › Figure S3.jpg]

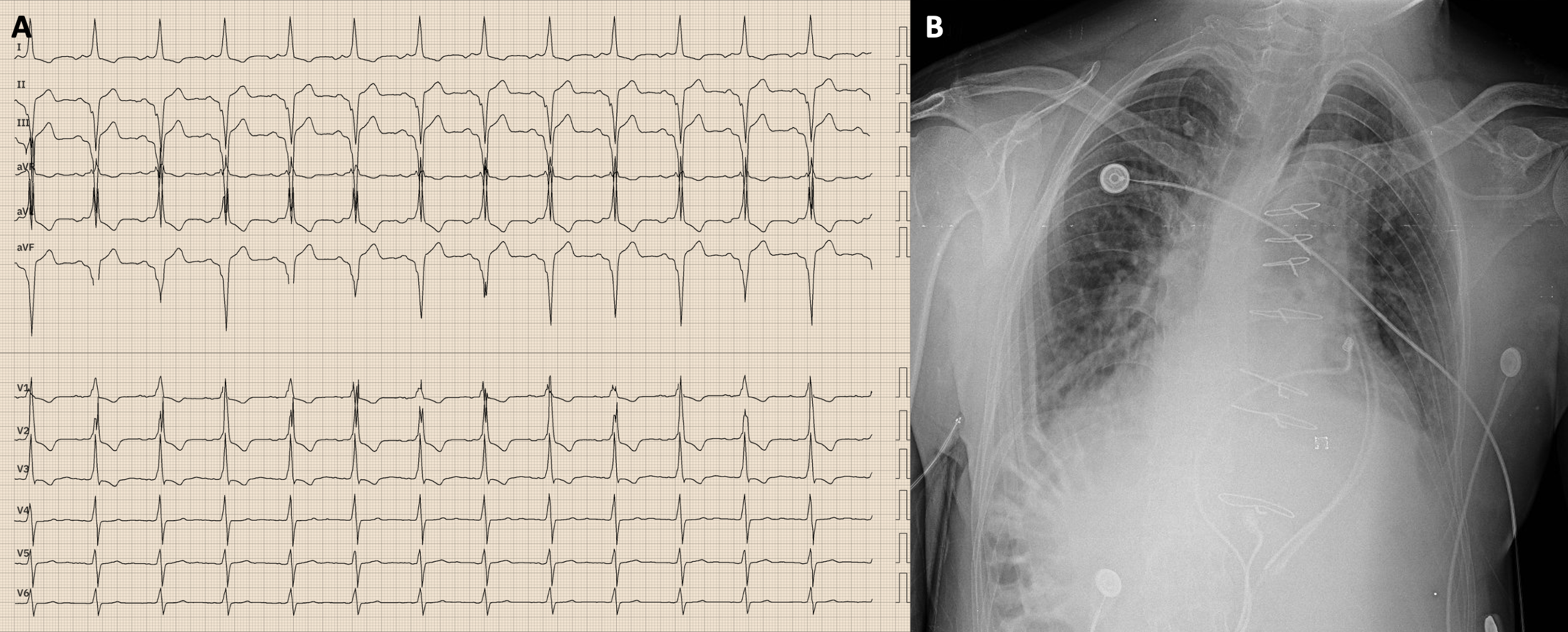

Supplement: ytae666_Supplementary_Data [file ytae666_supplementary_data.zip › Figure S4.jpg]
